# PCA-based supervised identification of biological soil crusts in multispectral images

**DOI:** 10.1016/j.mex.2019.03.013

**Published:** 2019-03-20

**Authors:** Thomas Fischer

**Affiliations:** Brandenburg University of Technology Cottbus-Senftenberg, Central Analytical Laboratory, Konrad-Wachsmann-Allee 6, 03046 Cottbus, Germany

**Keywords:** PCA-based supervised identification of biological soil crusts in multispectral images, Multimodal imaging, Identification, Classification

## Abstract

It was the aim of the method development to classify types of various biological soil crusts (biocrusts) using principle component analysis (PCA) on multispectral images. To address this aim, visible (RGB) and NIR images of bare sandy soil, algal and moss biocrusts were registered, per channel reflection values were determined using a calibration color chart on a pixel basis, and a PCA was performed on the unfolded RGB-NIR reflectance hypercubes (i.e. three-dimensional hypercubes were transformed into x.y × λ 2D-matrices with λ channels serving as variables for PCA). The classification approach was based on the hypothesis that biocrust types map specifically in PCA ordination plots, meaning that distinct regions in ordination plots may be assigned specifically to individual biocrust types. Reallocation of the pixels assigned to biocrust types to their respective image coordinates would then yield biocrust classification plots.

• Allows manual selection of features or identification of given features in PCA ordination plots.
• Fully permits the selection of relevant and omission of irrelevant, as well as identification of unknown classes.
• It is not restricted to RGB-NIR multispectral data only, but may be applied to any type of multimodal imaging data.

## Graphical abstract

**Figure:**
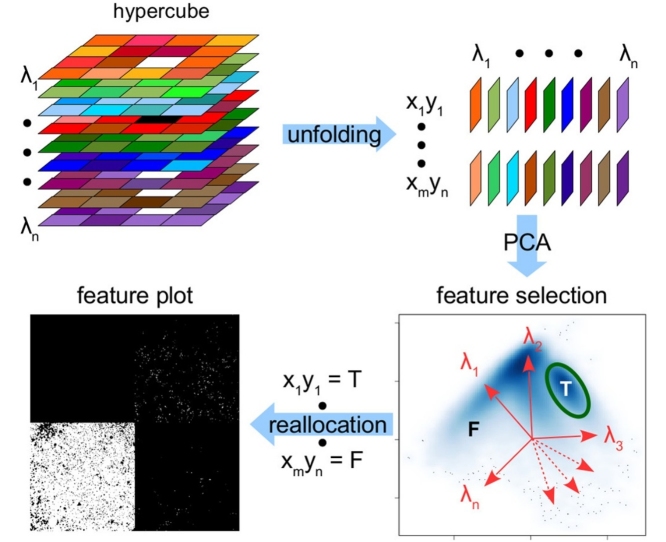

**Specifications Table**

**Table:** 

**Subject Area:**	• *Agricultural and Biological Sciences*
**More specific subject area:**	*Classification of biological soil crust types*
**Method name:**	*PCA-based Supervised Identification of Biological Soil Crusts in Multispectral Images*
**Name and reference of original method:**	*C. Rodarmel, J. Shan, Principal component analysis for hyperspectral image classification, Surveying and Land Information Science. 62 (2002) 115–122.*
**Resource availability:**	*example image files provided as supplementary material*

## Method details

The multispectral approach in remote sensing typically includes the estimation of spectral indices. For biocrusts, the normalized difference vegetation index (NDVI [1,2]) has been proposed; however, high NDVI values of wet biocrusts may be misinterpreted as vascular plant vegetation dynamics whereas dry biocrusts only gained negligible NDVI values [3]. To overcome this disadvantage, several more specific spectral indices have been proposed for biocrusts, like the crust index (CI, respectively [4]), the brightness index (BI [5]) or the biological soil crust index (BSCI [6]), where biocrusts are characterized by typical ranges of respective index values. All spectral indices well reflect crust activity or biomass but are not specific enough to differentiate between different crust types, like lichen, cyanobacterial, algal or moss biocrusts. Rodriguez-Caballero et al. [7] propose support vector machines (SVM) for supervised classification of biocrusts from hyperspectral remote sensing data. However, a common problem with linear spectral mixture analysis (SMA) remains when the number of spectral endmembers is greater than the number actually required to unmix an individual pixel in the scene [3]. Non-spectral unsupervised principal component analysis (PCA) classification of biocrusts has indicated that the development of the microbial community was affected at multiple scales, including biocrust successional stage, seasonal effect and the micro-geomorphology [8]. High-resolution VIS-NIR spectroscopy was employed to study the influence of wetting on cyanolichen-dominated biocrusts in a non-imaging approach [7]. There was no attempt so far in the literature to use multispectral PCA classification of biocrusts in high-resolution images.

The method proposed refers to Rodarmel and Shan [9] who suggested principal component analysis for preprocessing of hyperspectral images. Based on their finding that only the first few principal component image bands contain significant information, this study aimed at elucidating the feasibility of biocrust classification by manual selection of spectral features in PCA ordination plots.

**Fig. 1:**
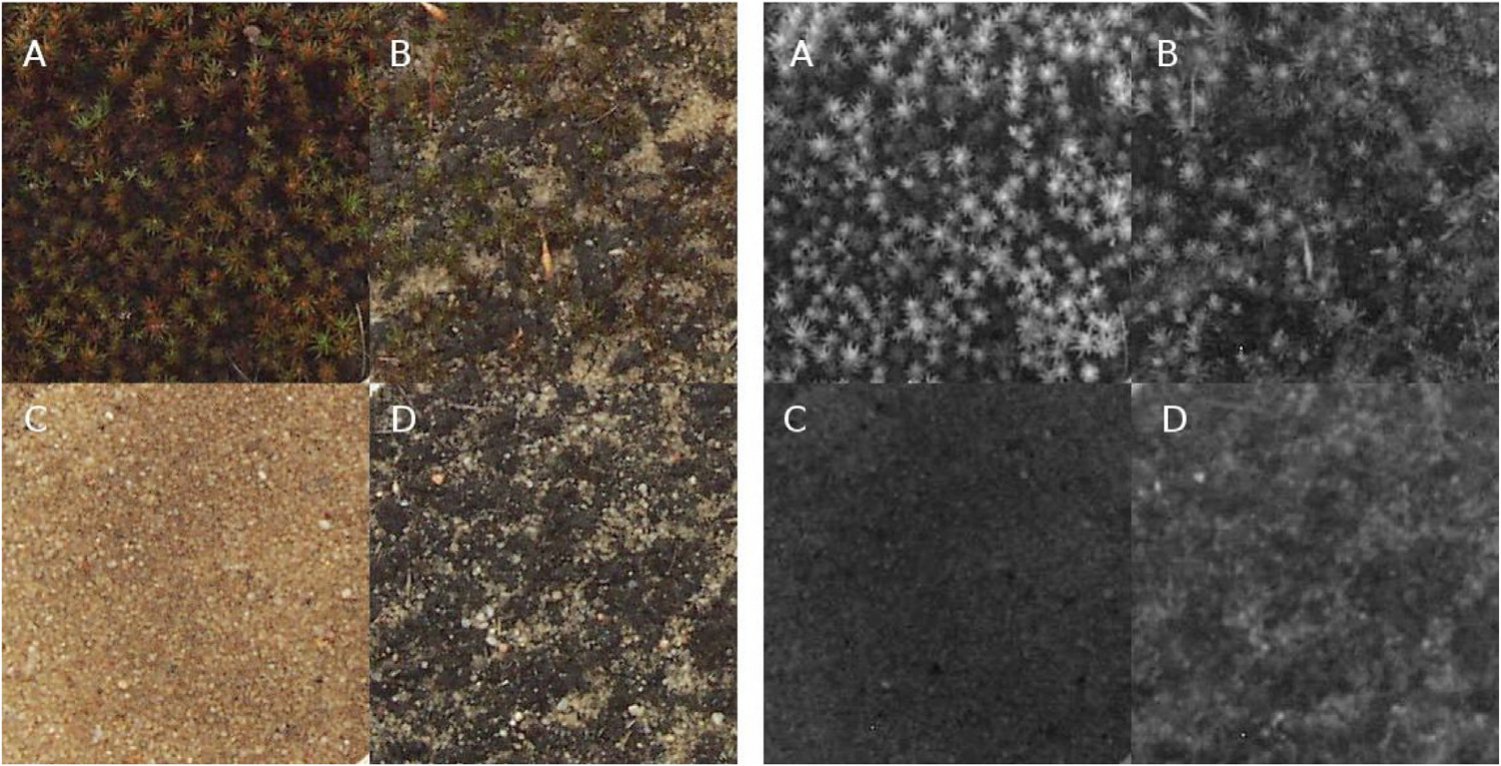
RGB (left) and NIR (right) images used for biocrust classification. A – Polytrichum-dominated moss biocrust, B – mixed biocrust, C – bare sandy soil, D – Zygogonium-dominated algal biocrust selected.

The study site was a catena from the mobile part of an inland dune to dry acidic grassland dominated by *Corynephorus canescens* and located near Lieberose, Brandenburg, northeast Germany (51°55′49″N, 14°22′22″E). A detailed description of the sampling site is given by [10]. The samples represented the sandy substrate, an algal biocrust dominated by *Zygogonium ericetorum*, a moss crust dominated by *Polytrichum piliferum*, and a mixed biocrust composed of both *Z. ericetorum* and *P. piliferum* (Fig. 1).

### Method workflow

**Fig. 2:**
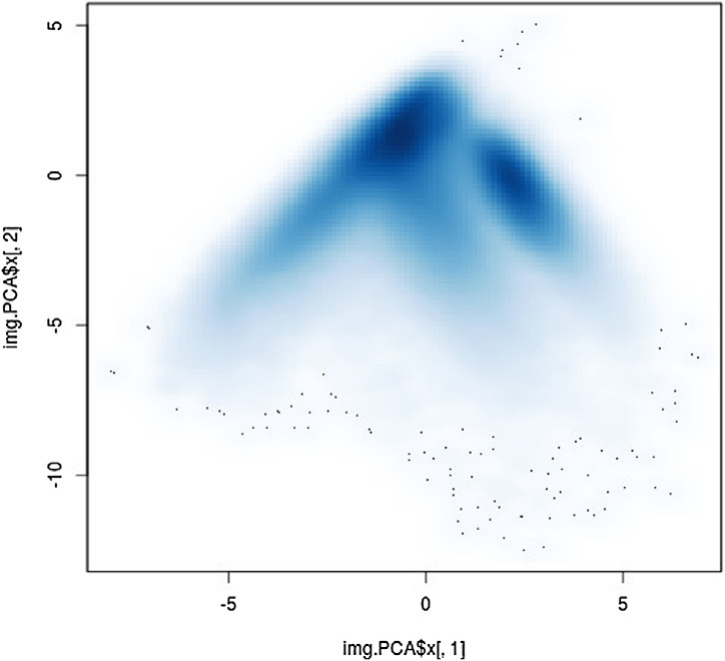
PCA ordination density plot of the first and second components of unfolded hypercube.
6 Select spectral features by outlining a polygon with the mouse pointer. The function „fhs “(freehand select) of the gatepoints package allows selection of data points by outlining polygons in two-dimensional scatterplots. In the present method, each data point represents spectrally identical pixels in a PC1 vs. PC2 density plot, where data points inside the polygon were denoted as „T “(TRUE) and all other data points as „F “(FALSE). Close selection with click on right mouse button and plot selection result using the previously defined plot_PCA function (see step 1 of this section), which generates binarized plots with selected features being represented in white color and all other in black. Fig. 3 shows the selection of the data points representing the bare sandy soil, algae and mosses.
  selectedPoints <- fhs(kk2, names = F, pch = NA_integer_)
  plot_PCA(selectedPoints)

1 Load packages gatepoints and jpeg of the R software suite [11]. Define two functions to plot image objects (plot_jpeg) and to reallocate the result of classification to image coordinates (plot_PCA). The R code for the two functions is listed in the additional information section.
2 Load the RGB and the respective registered NIR image files into the workspace and store the xy-dimensions in variable „res“, where imageNIR-reg.jpg and imageRGB.jpg denote the calibrated and registered NIR and RGB image files.
  imgRGB.stat <- readJPEG("imageRGB.jpg") # load RGB image
  imgNIR.stat <- readJPEG("imageNIR-reg.jpg") # load NIR image
  res = dim(imgRGB.stat)[1:2]
3 Merge image objects to hypercube denoted as „imgMult“. The number of spectral channels (the z-dimension) of the hypercube equals sum of the spectral channels of the RGB and NIR images. Unfold hypercube to object „imgMult.1d“.
  imgMult <- array(c(imgRGB.stat[, , 1:3], imgNIR.stat[, , 1:3]), dim = c(res[1], res[2], (dim(imgRGB.stat)[3] + dim(imgNIR.stat)[3])))
  imgMult.1d <- array(imgMult, dim = c(res[1]*res[2], (dim(imgRGB.stat)[3] + dim(imgNIR.stat)[3])))
4 Run PCA. For the given example images, the first two components explained 9615% of the total variance.
  img.PCA <- prcomp(imgMult.1d, scale. = T)
5 Create empty dataframe for selection results (kk2) and PC1 vs. PC2 density plot. Each coordinate in Fig. 2 represents a single spectrum, where clusters of high pixel density denote objects with similar spectral features.
  kk2 <- data.frame(img.PCA$x[,1], img.PCA$x[,2])
  smoothScatter(img.PCA$x[,1], img.PCA$x[,2])

**Fig. 3:**
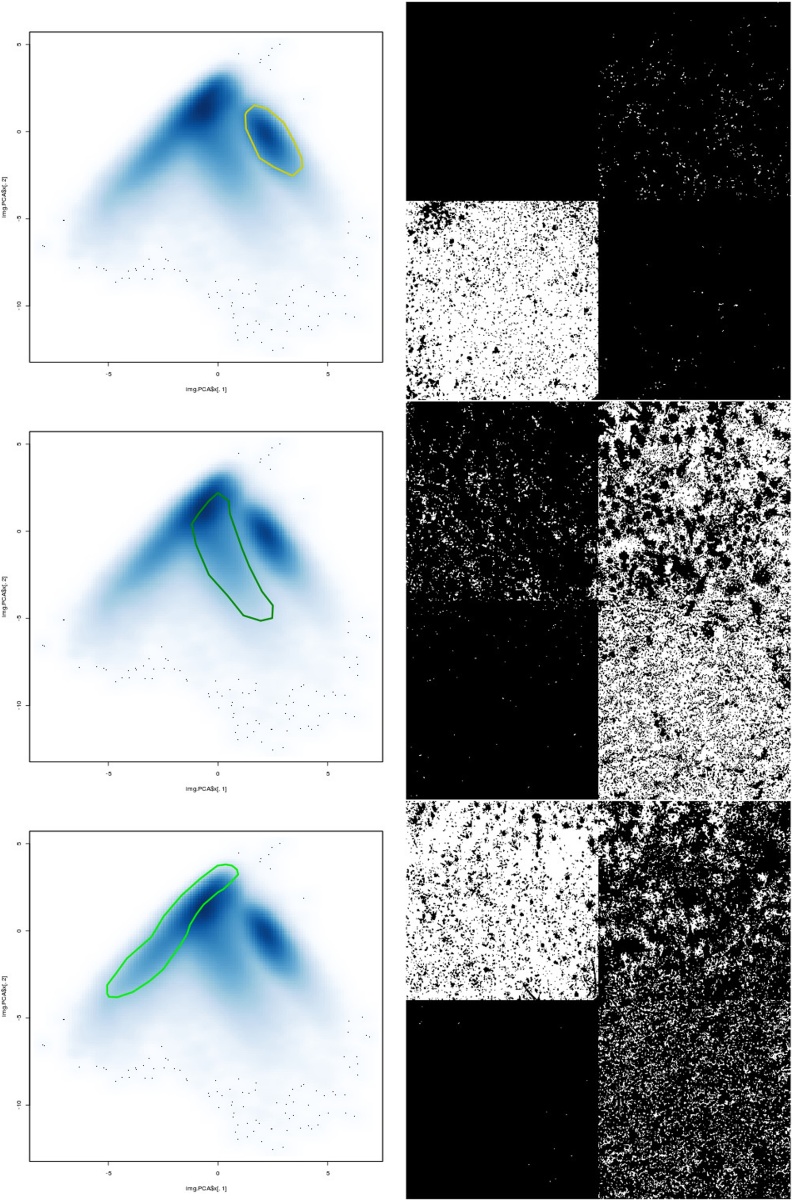
The data points outlined by yellow, dark and bright green polygons (left) represent pixels of bare sandy soil, as well as algae and mosses in the biocrusts, respectively (right).

### Hint 1

**Fig. 4:**
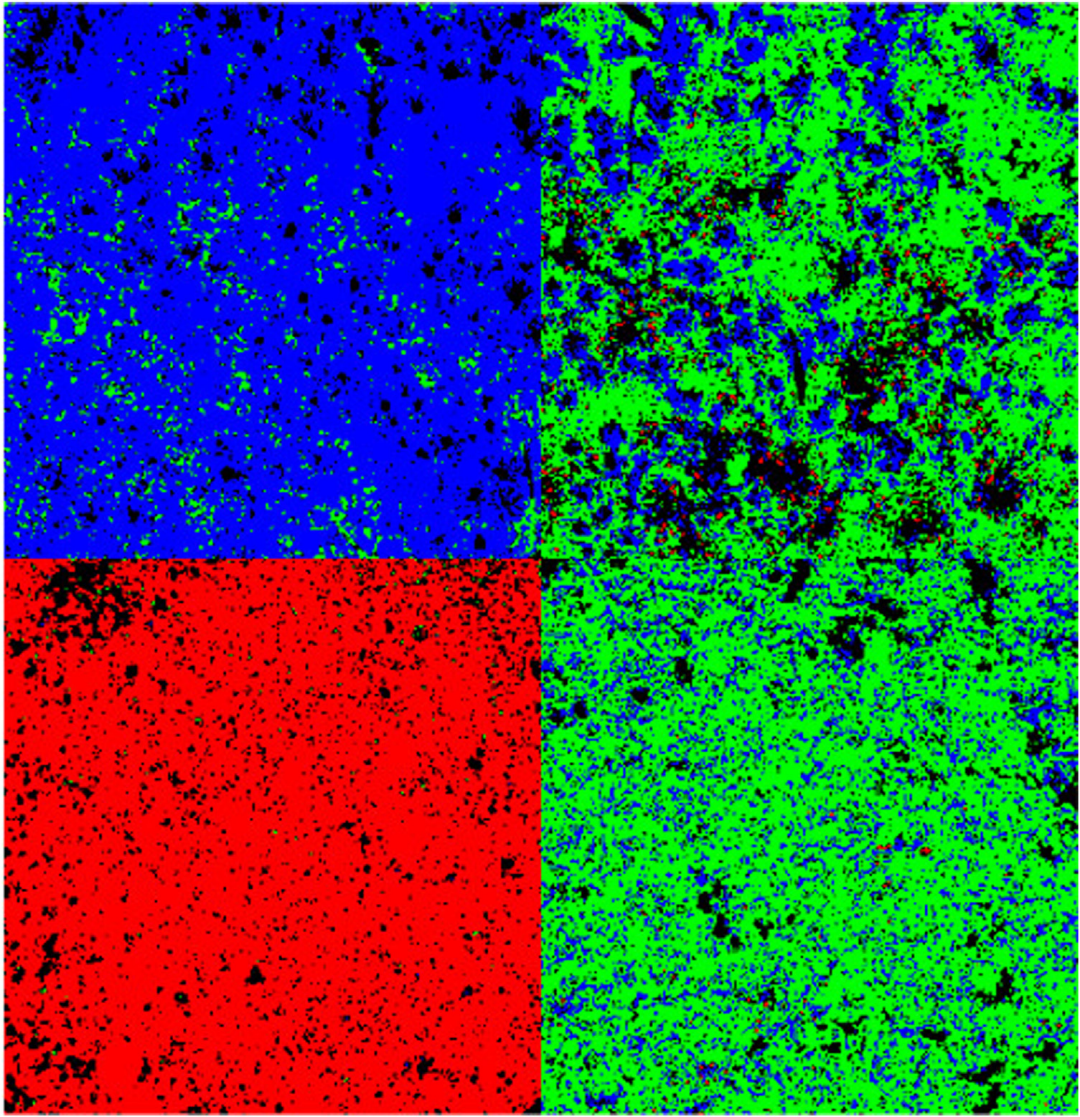
Biocrust classification image. Red – bare sandy soil, green – algae, blue – mosses.

Step 6 may be repeated multiple times and allows the simultaneous presentation of multiple classes, for example in combined color images of biocrust types (use, for example, selectedPoints1, selectedPoints2 etc. as fhs output for multiple polygons, Fig. 4).

### Hint 2

**Fig. 5:**
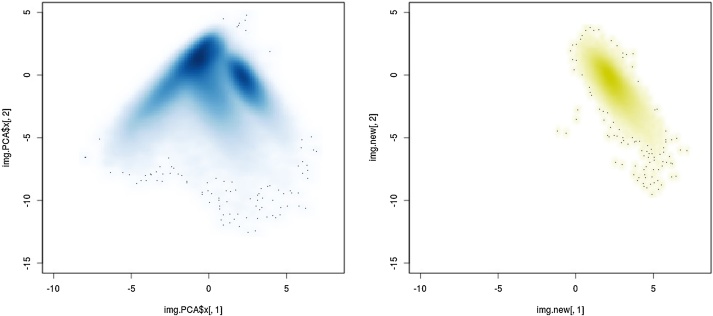
PCA ordination density plot of unfolded hypercube of all biocrust types (left) and of the bare sandy soil only (right).

Situations may arise when it is necessary to identify the area where objects with given spectral features are represented in the ordination plot. The „predict “function of R may be deployed in this case with steps 2 and 3 of the method workflow being performed for an image containing the desired objects only. Fig. 5 depicts the result of the projection of pixels representing bare sandy soil only onto the PCA space generated in step 4. The prediction results were stored in the variable “img.new”.

img.new <- predict(img.PCA, newdata = bareSand.1d)

smoothScatter(img.PCA$x[,1], img.PCA$x[,2], xlim = c(-10,8), ylim = c(-15,5)) # Fig. 5 left

smoothScatter(img.new[,1], img.new[,2], colramp = colorRampPalette(c("white", "yellow3")),

xlim = c(-10,8), ylim = c(-15,5)) # Fig. 5 right

### Hint 3

The binarized plots of biocrust types may be used for further image analysis, like calculation of coverages (Fig. 7) or for geostatistical analyses.

### Example

**Fig. 6:**
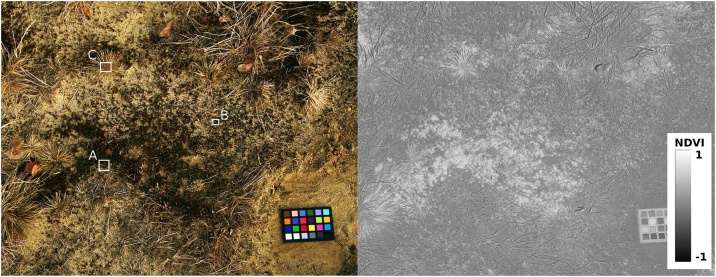
RGB (left) and NDVI images (right) of an example spot covered with algae, mosses and grass. The training spots for projection of spectral features onto the PCA space (see HINT 2) are outlined as A (mosses), B (algae) and C (grass). A high-resolution version of the image is available as eSlide.

Provided that the normalized difference vegetation index (NDVI) correlates with net primary production [12], the proposed approach may be used to estimate the relative contribution of biocrust types to photosynthesis. Fig. 6 depicts an example spot covered with an algae, mosses and some higher vegetation (grass), as well as the NDVI image of that spot.

**Fig. 7:**
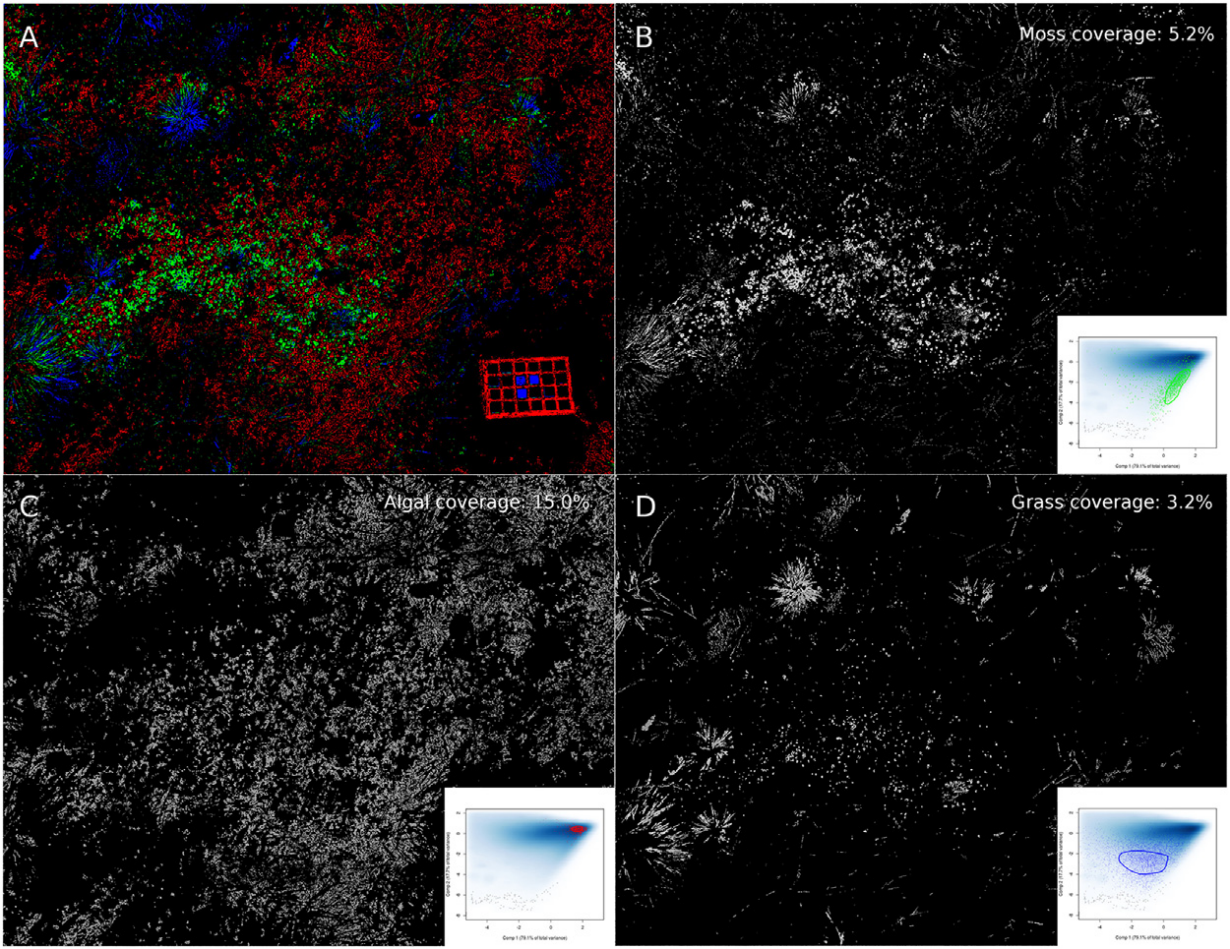
Combined biocrust type image (A) with mosses (green), algae (red) and grass (blue); NDVI images of mosses (B), algae (C) and grass (D). The PCA ordination plot, data points of training spots (see Fig. 6) and the polygon for manual feature selection for each biocrust type are shown in the lower right corners. Coverages were estimated according to HINT 3. Trampled areas and color checker were disregarded for analysis. A high-resolution version of the image is available as eSlide.

**Fig. 8:**
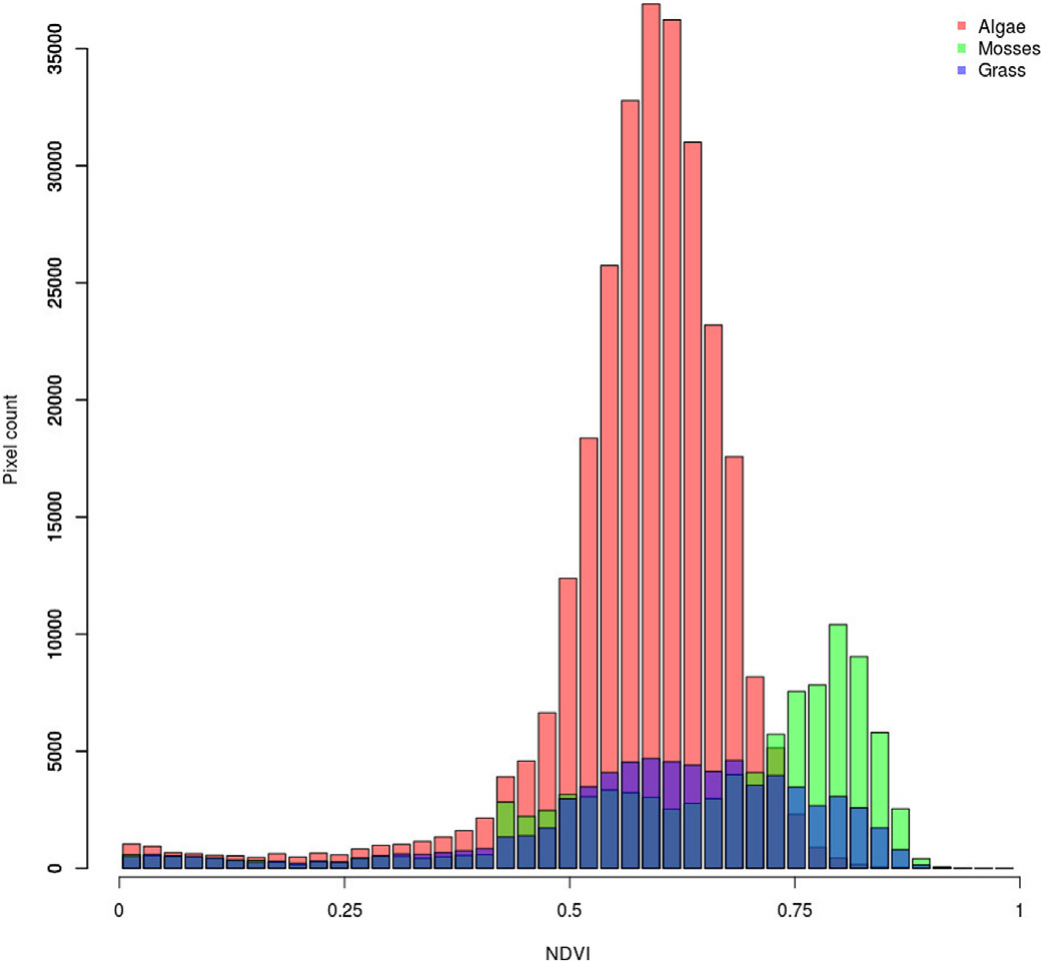
NDVI histograms of mosses, algae and grass estimated from Fig. 7B–D, respectively. Relative histogram areas represent the contribution of individual biocrust types to total photosynthesis. In the given example, mosses, algae and grass contributed with 21.4, 62.5 and 16.1%, respectively, to total photosynthesis.

The NDVI image was subsequently masked with binary images of mosses, algae and grass, where the pixel values of 255 were set to full transparency (Fig. 7). Then, the pixel value histogram of the resulting combined images would represent the distribution of net primary production for each biocrust type (Fig. 8).

## Figures and Tables

**Figure:**
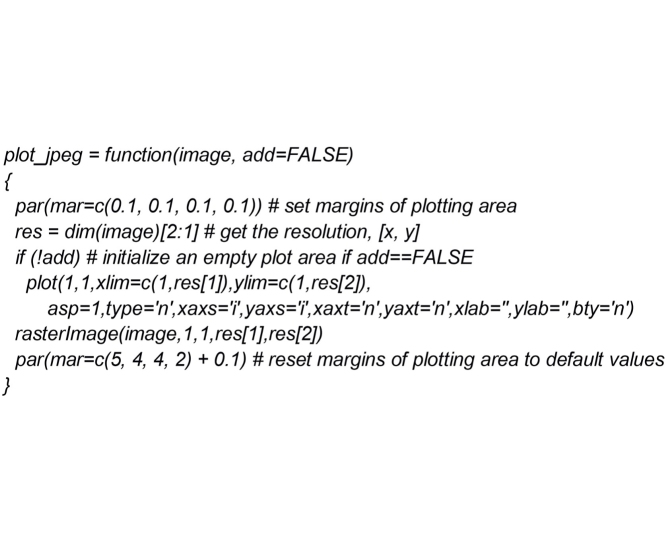


**Figure:**
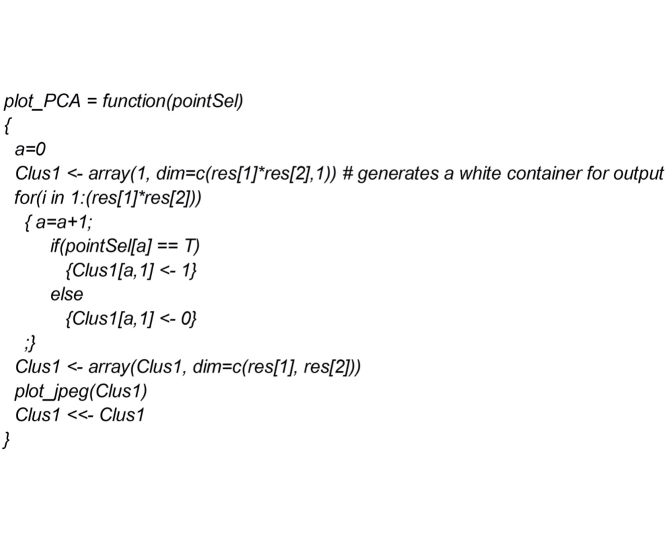


## Additional information

The method presented is the combined outcome of a biocrust-related ecological project and a Horizon2020 COST action on multimodal imaging in forensic science. Several methods for object classification were tested, where particular emphasis was put on recognition of unknown classes and handling of irrelevant „unclassifiable“ objects. It was found that supervised techniques (random forest, linear discriminant analysis (LDA) and support vector machines (SVM) were tested), which are unable to classify unknown classes by their nature, assigned irrelevant objects to one of the given classes of relevant objects. Unsupervised techniques, like k-means clustering, require the definition of the amount of classes to be separated, which bears a given risk of over- or underestimation of class numbers. This means that objects belonging to one class may be split into several classes (overestimation) or that objects belonging to several classes are merged into one (underestimation), where no distinction between relevant or irrelevant objects is made. The method proposed allows the manual selection of features or the identification of given features in PCA ordination plots, which fully permits the selection of relevant and omission of irrelevant objects, as well as identification of unknown classes. I present the biocrust branch of the method development here, because it gives the essence of the method in a nutshell. The forensic branch, the nondestructive recognition of bodily fluids, requires spectral preprocessing to account for the influence of the carrier material, which needs to be addressed in a separate study.

R code for function “plot_jpeg” to plot image objects, required for plot_PCA (see below)

R code for function “plot_PCA” to plot the output of the pixel selection function

### Image registration workflow

VIS and NIR photographs were taken using a Olympus Camedia Z5000 consumer camera. Reflectance calibration of the images was performed using a ColorChecker classic (X-Rite) with known RGB and NIR reflectance values of all 24 chart cells [12]. Deviating from [12], the following MATLAB v2018b procedure (The MathWorks, Inc.) was used for image registration, where imageNIR and imageRGB denote the imported MATLAB image objects from NIR and RGB image files, respectively, into the workspace.

1 Select pairs of control points, copy control points as movingPoints for the NIR image and fixedPoints for the RGB image to workspace when finished. This means that the NIR image will be aligned to fully match the RGB image. A minimum of 8 control point pairs yielded best results.
  > cpselect(imageNIR, imageRGB)

2 Infer spatial transformation from control point pairs. „t_concord“ contains the transformation matrix for the NIR image object and will be used later in step 4.
  > t_concord = cp2tform(movingPoints,fixedPoints,'projective');

3 Retrieve information about the RGB file. The dimensions of the image file „imageRGB.jpg“ are stored into the object „info“ and will be used to correctly fit the registered NIR image to correct image size in the next step.
  > info = imfinfo('imageRGB.jpg')

4 Transformation of NIR image object to align with RGB image. Transformed image object „registered“ is saved as image file „imageNIR-reg.jpg“.
  > registered = imtransform(imageNIR ,t_concord, 'XData',[1 info.Width], 'YData',[1 info.Height]);
  > imwrite(registered, 'imageNIR-reg.jpg');
